# Utility and usability of a dengue NS1 rapid diagnostic as a self-test

**DOI:** 10.1186/s41182-025-00705-9

**Published:** 2025-02-24

**Authors:** Nurfatihah Zulkifli, Naim Che-Kamaruddin, Atiqah Hazan, Tan Kim-Kee, Sivalingam Rajagopal, Sazaly AbuBakar

**Affiliations:** 1 Tropical Infectious Diseases Research and Education Centre (TIDREC), Level 2, High Impact Research (HIR) Building, Universiti Malaya, 50603 Kuala Lumpur, Malaysia; 2 Accobiotech Sdn. Bhd. Industrial Park, 81750 Masai, Johor Malaysia

**Keywords:** Infectious diseases, Arboviruses, Dengue virus, NS1, Self-test, Utility, Usability

## Abstract

**Background:**

Early detection of dengue fever is pivotal to help differentiate against other febrile illnesses, especially in the dengue-endemic regions. Currently, febrile patients would have to go to the healthcare facility to get tested for dengue. A diagnostic approach that empowers febrile persons to perform their own tests is needed. Here, we evaluated the utility and the usability of the dengue NS1 rapid diagnostic test and whether it could be accepted as a home self-test. A lateral flow immunochromatography test (ICT) using DENV NS1 was converted to a possible self-test kit by providing the kit with a test device, an alcohol swab, a lancet, a disposable dropper, an assay buffer, and a test manual.

**Methods:**

Twenty volunteers were recruited for this study. The participants performed the self-test under the observation of trained observers who recorded if any procedural error was committed. The results of each test were interpreted by the participants using a given interpretation table.

**Results:**

Ninety-five percent (95%) of the study participants found the instruction manual was easy to follow and 70% felt the test kit was easy to use. Additionally, 80% of the participants successfully obtained the expected results. A majority (80%) would consider using the test kit if needed and would recommend it to family members and friends. Seventy percent (70%) of the participants, most of whom with monthly income of less than USD417, were willing to pay less than USD2 for the test kit.

**Conclusion:**

Findings from the study suggested that a self-test diagnostic for dengue fever is highly acceptable and, hence, could be a viable approach for the early detection of the infection.

*Trial registration* MRECID.NO: 2022628-11345.

## Background

Dengue fever usually manifests as a non-specific febrile illness and is often associated with fever, joint pain, headache, and malaise, which could resemble the early symptoms of other arboviruses infections [20, 21, 27], bacterial infections [4, 8], rodent-borne virus diseases [12] and COVID-19 [3, 14]. The continuous presence of severe acute respiratory syndrome coronavirus 2 (SARS-CoV-2) infection in the population has complicated the diagnosis of dengue in most dengue-endemic regions [1, 26]. While treatment for dengue fever is mainly supportive to ameliorate the more prominent symptoms and reduce the chances for the development of complications and organ impairments, specific treatment for dengue has yet to be identified [24].

Several candidate dengue vaccines are available, but none of the currently available vaccines are broadly recommended for those living in areas where dengue is endemic. At the moment, in most areas where dengue is endemic, which often also coincides with areas where the population is economically disadvantaged, febrile individuals would need to go to the healthcare facility to get tested. This could be a challenge as the healthcare facilities could be at a distance, making traveling inconvenient due to transport and accessibility issues. Additionally, unwell individuals who rely on daily wages would have to take time off from work to visit the hospital for treatment, thus risking disrupting work productivity and losing income.

Due to these inconveniences, many would delay seeking medical attention and refrain from going to the hospital until the symptoms have worsened and this has been strongly linked to severe dengue complications [25]. Hence, a diagnostic approach that would empower a febrile person to perform a self-test could be a potential alternative [18]. Results obtained from an early detection of an infection could help the person to seek early treatment, enabling the medical practitioner to effectively address the affected individuals. Conventionally, diagnosis of dengue is mainly done by clinicians who assess the patient’s history and the clinical presentation. Confirmation of the diagnosis includes antibody testing for dengue IgM and/or IgG, but these tests are often labor-intensive, time-consuming, and require specialized expertise and expensive equipment. The introduction of the dengue virus non-structural protein 1 (NS1) rapid diagnostic test, however, has simplified the testing, making it widely accessible to most healthcare facilities [13]. This test, however, is still performed by professionals in healthcare facilities.

The use of rapid diagnostic tests for infectious diseases was proven essential at the population level, especially during the COVID-19 pandemic, where the availability of a rapid diagnostic test kit was expanded beyond the hospital confines, making it highly accessible to the public at large. Individuals who tested positive for COVID-19 using the home self-test were advised to adhere to self-isolation protocols [23]. This highlights the importance of early detection of the infection in mitigating onward virus transmission [17, 23]. A parallel diagnostic approach could be adopted for dengue, potentially offering similar benefits in disease management and containment efforts which many were already expressing their intentions to use if it becomes widely available [30]. A rapid diagnostic test based on the detection of NS1 antigen, which is detectable up to 11 days after the onset of fever but peaks on day 6 [5, 16, 29] has been developed and widely used. Many earlier studies have also shown that a higher NS1 serum level was linked to the severe manifestations of dengue [11]. Severe dengue and deaths, hence, could be avoided if the status of the patient is known early through the detection of the NS1 antigen.

In the present study, a diagnostic test based on dengue virus NS1 antigen-capture immunochromatographic assay was converted into a possible self-test kit. Here, we described an evaluation of the dengue NS1 rapid diagnostic test for the detection of dengue fever for its utility and usability as a potential out-of-healthcare facility self-test kit.

## Methods

### Statement of ethics approval

The study received the Institutional Review Board (IRB) ethics approval (MRECID.NO: 2022628-11345) from the Medical Research Ethics Committee (MREC), Universiti Malaya Medical Centre (UMMC).

### Study design

**Fig. 1:**
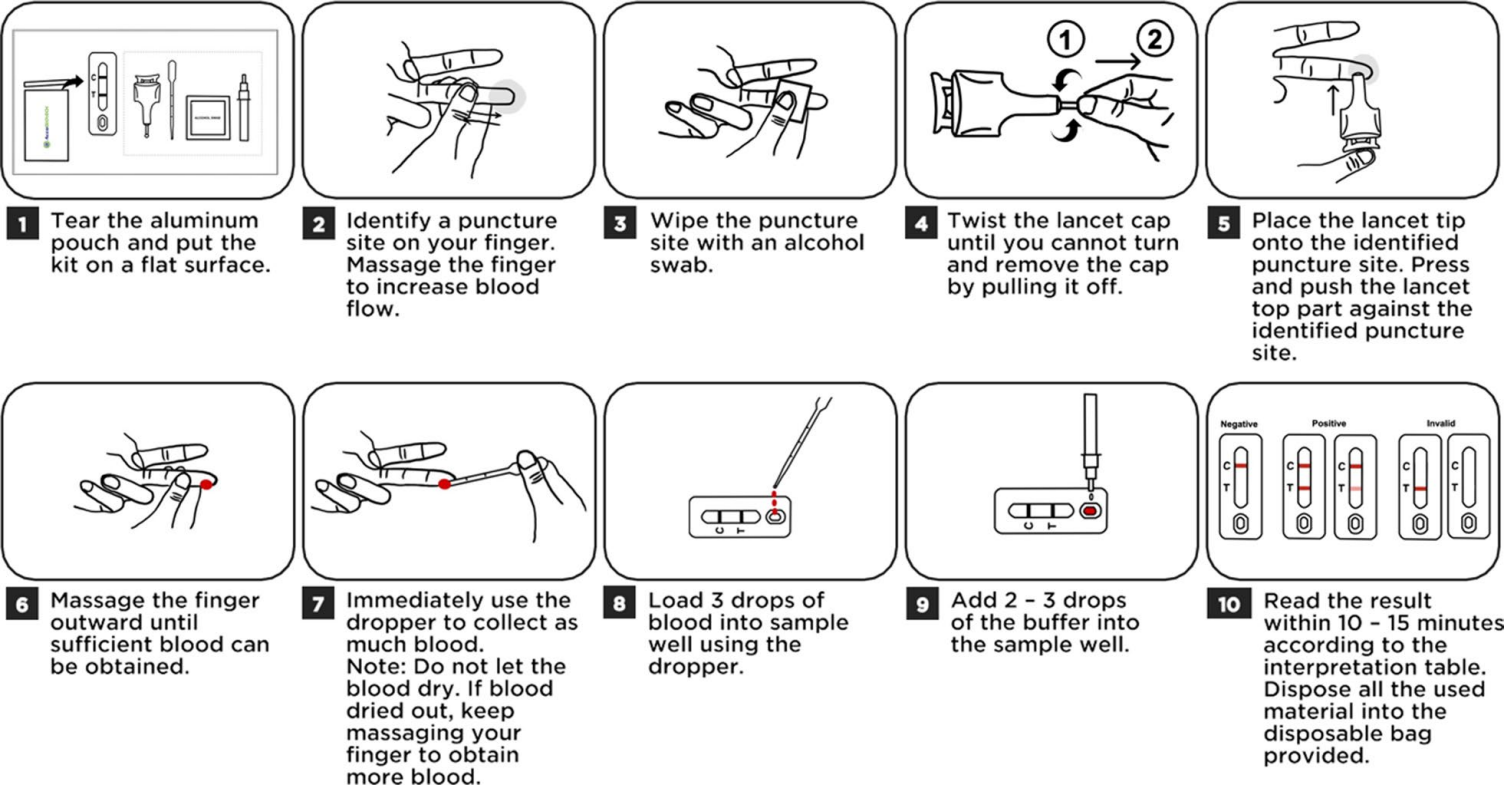
Schematic drawing of the instruction manual provided in the self-test kit

Prior to conducting the study, half of the sample lysis buffer in the test kit was spiked with a known concentration of recombinant dengue virus NS1, while the other half served as a placebo with nothing added. The test kits were given out randomly to the participants and they were not informed about the kit’s composition until after completing the test. This study was conducted involving two parties (the observer and the volunteer). The observer was first trained to familiarize themselves with the study protocol and workflow. A simulation of the study was conducted to ensure that the observers were adequately prepared to accurately perform the study. A pre-test questionnaire to assess the participants’ previous laboratory experiences was given to participants before conducting the test. Participants were given the Dengue NS1 Ag Home Test kit (AccoDengue® Home Test, AccoBiotech Sdn. Bhd., Malaysia) consisting of the reagents, a test device, a single-use disposable lancet, a disposable dropper, and an instruction manual in the form of written text and a QR code, which directs to an instruction video (Fig. 1). The time taken for the participants to perform the test and the activity were recorded by an observer who recorded the activities in an evaluation form. Upon completion, the test device was collected and the results were digitalized and read by the experts. Participants were given a post-test questionnaire to assess their satisfaction and experience while performing the test. Data were collected, stored, and analyzed by the principal investigator.

### Recruitment of participants

The sample size of this study was determined based on previous studies [6, 19]. Twenty participants were randomly recruited from among the members of Universiti Malaya (UM). This sample size would give an estimated 95% of the usability problems in the self-test kit design [6]. Recruitment of participants for the study was done by advertising the study on the UM Bulletin Boards, social media platforms, physical advertisements in the faculties, and through the UM email system. The first twenty qualified volunteers were selected based on the inclusion criteria shown in Table 1. The study details were explained to the volunteers and an informed consent form was provided. Participation was voluntary and they could withdraw from the study at any time. Participants were nominally compensated for their hardship and inconvenience considering the minimal travel costs as we targeted the public members of the UM and the evaluation process was less than an hour.

**Table 1:** Inclusion criteria for the recruitment of participants

Inclusion criteria	
1. Healthy individuals 2. Aged 18 to 45 years old 3. Give voluntary informed consent to participate in this study	

### Data analysis

Data were tabulated and analyzed using Microsoft Excel (Microsoft, Washington, USA). Descriptive analysis was performed to describe and summarize the data. Kruskal–Wallis non-parametric test was performed to determine the significant differences between the participants with different years of laboratory experience against the number of deviations from the instruction manual. The test was also utilized to calculate the significant differences of groups with different numbers of deviations from the instruction manual and different years of laboratory experience against the accuracy of the results.

## Results

### Demographic characteristics of the study participants

A total of twenty participants who had never performed the dengue NS1 rapid diagnostic test were recruited for this study. The participants were grouped into three according to whether they had any laboratory experience and the extent of their experience. Based on the participants’ research experience, 35% of the participants had no experience working in the laboratory, 25% spent less than 5 years working in the laboratory, while the remaining 40% spent more than 5 years working in the laboratory setting (Table 2). All participants completed the test within the suggested time of 10 min. The average age of the participants was 33.75 ± 6.05 SD, whereby half of them (50%) were male. The gross monthly income was used as a surrogate indicator of the level of education [7].

**Table 2:** Demographic characteristics of the study participants

Demographic	Number (%)
Age	
18–20 years old	0
21–30 years old	5 (25)
31–40 years old	11 (55)
41–45 years old	4 (20)
Gender	
Male	10 (50)
Female	10 (50)
Gross monthly income	
≤ MYR 2,000 (~ USD400)	9 (45)
≤ MYR 3,000 (~ USD625)	3 (15)
≤ MYR 5,000 (~ USD1050)	7 (35)
≤ MYR 10,000 (~ USD2100)	1 (5)
Have you worked in a laboratory?	
No	7 (35)
Yes	13 (65)
Have many years of laboratory experience have you had?	
≤ 5 years	5 (25)
≥ 5 years	8 (40)
How much laboratory work did you do each month?	
Less than once a week	0
1–2 times a week	4 (30.77)
3–5 times a week	5 (38.46)
More than 5 times a week	4 (30.77)
Do you routinely read the manual before performing your work?	
Yes, while doing the laboratory work	6 (46.15)
Yes, immediately before starting	6 (46.15)
Yes, the night before	3 (23.08)
Never, use experience and instincts	0

### Effectiveness of the instruction manual

The effectiveness of the given instruction manual for performing the test was evaluated through the post-test questionnaire. The instruction manual provided an overall introduction to the home test kit, an infographic of step-by-step procedures to perform the test, and a QR code to access a video version of the user manual. Out of the twenty participants, half of the participants strongly agreed with the manual being easy to follow, 45% (9/20) agreed while 5% remained neutral (Table 3). On the other hand, 45% (9/20), strongly agreed that the instruction manual was clear, complete, easy to understand, and easy to follow, an additional 45% (9/20) agreed with this description while 10% (2/20) remained undecided (Table 3). Only one of the participants used the QR code to watch the instructional video.

**Table 3:** Effectiveness of the dengue self-test kit instruction manual and participants' laboratory experiences

Comments	Number of participants (%)
The instruction sheet was easy to follow	
a. Strongly disagree	0
b. Disagree	0
c. Neutral	1 (5)
No laboratory experience	*1*
Less than 5 years of experience	*–*
More than 5 years of experience	*–*
d. Agree	9 (45)
No laboratory experience	*4*
Less than 5 years of experience	*1*
More than 5 years of experience	*4*
e. Strongly agree	10 (50)
No laboratory experience	*2*
Less than 5 years of experience	*4*
More than 5 years of experience	*4*
The instruction sheet was clear, complete, and easy to understand	
a. Strongly disagree	0
b. Disagree	0
c. Neutral	2 (10)
No laboratory experience	2
Less than 5 years of experience	–
More than 5 years of experience	–
d. Agree	9 (45)
No laboratory experience	*3*
Less than 5 years of experience	*1*
More than 5 years of experience	*5*
e. Strongly agree	9 (45)
No laboratory experience	*2*
Less than 5 years of experience	*4*
More than 5 years of experience	*3*

Deviations of whether or not the participants strictly adhered to the instruction manual were recorded by trained observers. There were seven distinct steps involved in assessing the deviation (Fig. 1). The majority, 65% (13/20) of the participants, completed the test with no deviation. The remaining of them, 35% (7/20), completed the test despite introducing a single deviation, but no error in the test reading was recorded (Table 4). There were no statistically significant differences (*p*-value = 0.985; Kruskal–Wallis; Table 4) between the groups of participants, irrespective of their laboratory experience.

**Table 4:** Number of participants that performed the dengue self-test with deviation(s) and their corresponding laboratory experiences

Details	Number of participants (%)
Number of participants that performed the test with 0 deviation	
a. No laboratory experience	5 (38.46)
b. Less than 5 years of experience	2 (15.38)
c. More than 5 years of experience	6 (46.13)
Number of participants that performed the test with 1 deviation	
a. No laboratory experience	2 (28.57)
b. Less than 5 years of experience	3 (42.86)
c. More than 5 years of experience	2 (28.57)

Type of analysis	*p*-value (Significance, *p* < 0.05)
Summary of Kruskal–Wallis non-parametric analysis	
Research experience versus number of deviations	0.985 (No)
Research experience versus result accuracy	0.368 (No)
Number of deviations versus result accuracy	0.766 (No)

### Ease of use

The assessment of the ease of use of the self-test kit was conducted through a post-test questionnaire. The results showed that 25% (5/20) of the participants strongly agreed that the test kit was easy to use, with 45% (9/20) agreeing with the statement. 10% (2/20) disagreed in which one of them had less than five years of laboratory experience and the other had more than five years of laboratory experience. The remaining of the participants remained undecided (Table 5). In response to the second inquiry, 30% (6/20) strongly expressed their willingness to use the test kit again, 50% (10/20) agreed with this statement while the remaining participants remained neutral (Table 5).

**Table 5:** The ease of use of the dengue self-test kit and participants' laboratory experiences

Comments	Number of participants (%)
I am able to use the test kit easily	
a) Strongly disagree	0
b) Disagree	2 (10)
No laboratory experience	*–*
Less than 5 years of experience	*1*
More than 5 years of experience	*1*
c) Neutral	4 (20)
No laboratory experience	*3*
Less than 5 years of experience	*–*
More than 5 years of experience	*1*
d) Agree	9 (45)
No laboratory experience	*2*
Less than 5 years of experience	*2*
More than 5 years of experience	*5*
e) Strongly agree	5 (25)
No laboratory experience	*2*
Less than 5 years of experience	*2*
More than 5 years of experience	*1*
I would use this kit again	
a. Strongly disagree	0
b. Disagree	0
c. Neutral	4 (20)
No laboratory experience	*1*
Less than 5 years of experience	*1*
More than 5 years of experience	2
d. Agree	10 (50)
No laboratory experience	*5*
Less than 5 years of experience	*2*
More than 5 years of experience	3
e. Strongly agree	6 (30)
No laboratory experience	*1*
Less than 5 years of experience	*2*
More than 5 years of experience	*3*

### Accuracy

The test kit’s accuracy relies on the study participant’s ability to obtain the expected results. The majority, 80% (16/20) of the participants managed to obtain the expected results irrespective of their laboratory experience. Twenty percent (4/20), however, failed to obtain the expected results (Table 6).

**Table 6:** Number of participants that managed to obtain expected results and their corresponding laboratory experiences

Details	Number of participants (%)
Number of participants that managed to obtain expected results	
a. No laboratory experience	5 (31.25)
b. Less than 5 years of experience	4 (25)
c. More than 5 years of experience	7 (43.75)
Number of participants that failed to obtain expected results	
a. No laboratory experience	2 (50)
b. Less than 5 years of experience	1 (25)
c. More than 5 years of experience	1 (25)

### Satisfaction

The participants’ satisfaction after trying the dengue self-test kit was evaluated. The results suggested that 20% strongly agreed they were satisfied with the test kit and 65% (13/20) agreed with the statement (Table 7). As for the second inquiry, 35% (7/20) strongly agreed and 45% (9/20) agreed to recommend the kit to their family and friends. The remaining of the participants remained undecided (Table 7). Toward the end of the questionnaire, participants were inquired about their willingness to pay for this kit. In Malaysia, the rapid antigen test typically costs USD10–USD20 for a pack of 10 tests. Seventy percent (70%) of participants were willing to pay for the self-test kit for less than USD2 (MYR10), with the majority (35%) coming from the lowest gross monthly income group. Another 20% of the participants were open to paying up to USD4 (MYR20) for the kit, while the remaining 10% were willing to pay USD6 (MYR30) for it (Table 8).

**Table 7:** Responses to queries about the satisfaction of using the self-test kit and their corresponding laboratory experiences

Comments	Number of participants (%)
I am satisfied with the kit	
a. Strongly disagree	0
b. Disagree	0
c. Neutral	3 (15)
No laboratory experience	*1*
Less than 5 years of experience	*–*
More than 5 years of experience	*2*
d. Agree	13 (65)
No laboratory experience	*5*
Less than 5 years of experience	*3*
More than 5 years of experience	*5*
e. Strongly agree	4 (20)
No laboratory experience	*1*
Less than 5 years of experience	*2*
More than 5 years of experience	*1*
I would recommend this kit to my family and friends	
a. Strongly disagree	0
b. Disagree	0
c. Neutral	4 (20)
No laboratory experience	*1*
Less than 5 years of experience	*1*
More than 5 years of experience	*2*
d. Agree	9 (45)
No laboratory experience	*4*
Less than 5 years of experience	*2*
More than 5 years of experience	*3*
e. Strongly agree	7 (35)
No laboratory experience	*2*
Less than 5 years of experience	*2*
More than 5 years of experience	*3*

**Table 8:** Participant’s willingness to pay for the dengue self-test kit and their corresponding gross monthly income

Comment	Number of participants (%)		
	≤ MYR 10 (~ USD2)	≤ MYR 20 (~ USD4)	≤ MYR 30 (~ USD6)
How much are you willing to pay for this kit?			
a. Gross monthly income ≤ MYR 2000	7 (35)	1 (5)	1 (5)
b. Gross monthly income ≤ MYR 3000	2 (10)	2 (10)	–
c. Gross monthly income ≤ MYR 5000	4 (20)	1 (5)	1 (5)
d. Gross monthly income ≤ MYR 10,000	1 (5)	–	–

## Discussion

This study represents an evaluation of the utility and usability of a potential self-test kit for the diagnosis of dengue fever. There are currently no specific treatments for dengue and patients rely mainly on supportive treatments. Early detection and rapid access to proper clinical treatment, however, could reduce the possibility of patients developing severe dengue. Self-test kits are a useful tool for screening infection rapidly and effectively identifying positive cases in the populations. Self-testing for infectious diseases became significant during the COVID-19 pandemic as a tool for early detection. Later in the pandemic, the self-test kits for the infection became readily available and easily accessible at pharmacies as over-the-counter (OTC) products. While COVID-19 has tragically claimed millions of lives worldwide, it has also highlighted the importance of increased public awareness in other areas including the utilization of self-test kits for detection of infectious diseases.

The present study emphasizes the utility and the usability of a rapid diagnostic test as a self-testing, involving participants with diverse laboratory backgrounds and demographics, under simulated conditions. Due to the limited resources and scope of the study, the current or the past dengue infection status of the participants was not determined. Additionally, the utility part of the study evaluates the functionality of the rapid diagnostic test when used as a self-test, while the usability part examines whether participants can successfully use it independently [28]. The dengue status of the participants, therefore, was not crucial for the study. In the present study, the sample size was determined based on Nielsen’s et al. [19] recommendation which suggested that five users are enough for usability testing which could reveal 80% of usability problems of the tested products. However, Faulkner et al. [6] suggested that additional users in the usability study could increase the odds of revealing the problems, thus allowing us to reassess the device or testing kits, if necessary, and implement modifications based on the findings. Hence, twenty participants recruited for the study would be sufficient to reveal a diverse range of usability issues.

The present study involved the use of readily available rapid diagnostic tests as a self-test. To use the test kit, the blood sample is obtained by a finger prick, which is similar to the widely used glucose monitoring devices. The self-monitoring of blood glucose (SMBG) allows diabetic patients to effectively monitor their conditions. They can without medical practitioners maintain glycemic control, decide on food intake, and identify and address hypoglycemia and hyperglycemia [10, 15]. This self-monitoring practice helps alleviate the burden for diabetic patients of having to go to the hospitals as frequently for blood testing. A similar self-testing approach for early detection of dengue, thus, could be adopted.

In the present study, the main objectives were to assess the ease of use, effectiveness of the instruction manual, ability to obtain accurate results, and satisfaction of users with the possible dengue self-test kit. Given their prior experience and presumed awareness of the COVID-19 self-test kits, it was anticipated that participants would have no difficulty using the test kit. Hence, it was not surprising that almost all the participants found the dengue self-test kit easy to use and were willing to use it again if needed. This sentiment was consistent among participants from diverse backgrounds, including those without prior laboratory experience. Most participants also agreed that the instruction manual, an essential element of the test kit’s utility, provided clear guidance that enabled them to perform the test accurately, consistent with findings from a study conducted by [2]. Here, our study participants successfully conducted the test based on the instruction manual provided. Even though an instructional video was provided, only one participant scanned the QR code and viewed the entire video before performing the test.

Two participants with the same laboratory backgrounds, however, encountered difficulties using the test kit, resulting in inaccurate results, which were not anticipated. The main reason for their failure was the difficulty in using the lancet, which affected the entire testing process. The lancets used were reported by three participants to be non-user-friendly. The difficulty in using the lancet was also previously highlighted in a study involving the use of HIV self-test. In that study, participants new to lancets improved their skill with a second lancet. Hence, multiple single-use lancets could be incorporated into the test kit for a better user experience [22]. In addition, introducing an improved, user-friendly lancet, featuring a finer and narrower needle, could potentially reduce discomfort and pain, especially for those who have fears of needles [2]. The depth of the needle penetration should also be taken into consideration to ensure sufficient blood is obtained while minimizing pain.

Incorrect interpretation of results was noted from a participant who otherwise accurately performed the test and this participant had no prior laboratory experience. While laboratory experience may help one interpret the results better, there were other participants without such experience who completed the test and accurately interpreted the results. Difficulty in interpreting self-test results, however, has also been highlighted for other self-tests [9, 22]. Suggestions, including using different symbols for Test and Control lines on the test cassette, have been noted to mitigate the issue [22]. These improvements could enhance the overall user experience and minimize the risk of misinterpreting results. Alternatively, these limitations could perhaps be overcome by emphasizing the usage of instructional video, supervised testing by trained assistants or volunteers, or online telemedicine.

The present utility and usability study for a dengue self-test kit highlights an opportunity to improve early dengue detection among febrile persons. Findings from the study suggest that the utility of the rapid diagnostic as a self-test was acceptable to the majority of the participants. However, the usability study revealed potential inconsistencies between individuals with different laboratory experiences, leading to wrong results interpretations and difficulty in conducting the test. Future studies involving supervised self-testing, either through telemedicine tools or in situ tests by professionals such as pharmacists, could overcome the current limitation of the use of the self-test kit. Additionally, dengue in many endemic countries primarily affects the children population. Despite the ease of use and convenience of utilizing the self-test kit as highlighted in the present study, the lack of involvement of children through parent–child pairs warrants further subsequent research.

## Conclusion

In a broader context, this study has shown the potential value of introducing a self-test kit for dengue as a complementary tool to existing diagnostic services available at most healthcare facilities. The increased awareness of self-test kits during the COVID-19 pandemic presents an opportunity to make rapid diagnostic tests for dengue accessible to the public in the comfort of their homes, thereby allowing early detection of dengue and enhancing public health efforts to contain the spread of dengue.

## Data Availability

No datasets were generated or analysed during the current study.

## Abbreviations

DENV: Dengue virus
ICT: Immunochromatography test
IRB: Institutional Review Board
LFIA: Lateral flow immunoassay
MREC: Medical Research Ethics Committee
NS1: Non-structural protein 1
OTC: Over-the-counter
SMBG: Self-monitoring of blood glucose
SARS-CoV-2: Severe acute respiratory syndrome coronavirus 2
UM: Universiti Malaya
UMMC: Universiti Malaya Medical Centre
